# Segmental bioelectrical impedance analysis can detect differences between the affected and non-affected limbs in individuals with hip osteoarthritis

**DOI:** 10.1186/s12891-023-06541-4

**Published:** 2023-05-25

**Authors:** Jocassia Silva Pinheiro, Filipe Ramos Carlos, Luis Carlos Caseiro-Filho, Celso Hermínio Ferraz Picado, Flávio Luís Garcia, Elaine Caldeira de Oliveira Guirro, Rinaldo Roberto de Jesus Guirro

**Affiliations:** 1 Laboratory of Physiotherapeutic Resources, Postgraduate Program in Rehabilitation and Functional Performance, Department of Health Sciences, Ribeirão Preto Medical School, University of São Paulo, Ribeirão Preto, SP Brazil; 2 Department of Orthopedics and Anesthesiology, Ribeirão Preto Medical School, University of São Paulo, Ribeirão Preto, SP Brazil

**Keywords:** Hip osteoarthritis, Electrical impedance, Phase angle

## Abstract

**Objective:**

To analyze the bioelectrical impedance parameters of the lower limbs of individuals with hip osteoarthritis and healthy individuals.

**Design:**

Cross-sectional study.

**Setting:**

The study was carried out at the Hip Surgery Outpatient Clinic.

**Participants:**

The volunteers had to be between 45 and 70 years of age, of both sexes, with a clinical and radiological diagnosis of hip osteoarthritis for at least three years, unilateral involvement, or a significant complaint in one hip.

**Methods:**

This was a cross-sectional study. Fifty-four individuals were recruited for the study, 31 individuals with hip osteoarthritis (OA group) and 29 healthy individuals for the control group (C group). Demographic and anthropometric data were collected and then the Numerical Pain Rating Scale, WOMAC, Harris Hip Score, and bioimpedance assessment were applied.

**Main outcome measure(s):**

Electrical bioimpedance parameters. Phase angle (PhA), impedance, reactance, and muscle mass.

**Results:**

There was a significant difference in phase angle (PhA), impedance, and muscle mass at 50 kHz frequency on the side affected by OA when compared to the contralateral side. In the OA group, there was a significant decrease in phase angle (PhA) -0.54 (-0.85 to -0.23) and muscle mass − 0.29 (-0.40 to -0,19), as well as an increase in impedance at the 50 kHz frequency on the side affected by OA when compared to contralateral side 21.71 (13.69 to 29.74). In the C group, there was no difference between the dominant and non-dominant sides (P > 0.05).

**Conclusion:**

The segmental electrical bioimpedance equipment can detect differences between limbs affected and unaffected by hip osteoarthritis.

## Introduction

Osteoarthritis of the hip (OA) is one of the most disabling and prevalent dysfunctions in the population [1]. It is a disease that affects various joint components and is characterized by the degeneration of articular cartilage.

Its diagnosis is commonly made through radiography and used to assess the amount of joint space narrowing, the presence of osteophytes and subchondral sclerosis or cysts, and magnetic resonance imaging, which assesses joint degeneration [2]. However, these methods have disadvantages because of the technique and costs involved.

In addition to imaging tests, self-report instruments, such as scales and questionnaires, are also used to aid in Diagnosis. The most widely used are the Lequesne Index of Severity for Osteoarthritis of the Hip (LISOH) [3] and the Western Ontario and McMaster Universities Osteoarthritis Index (WOMAC) [4]. Nevertheless, this type of evaluation uses a significant subjective component.

In this context, the use of electrical properties of body tissues has been investigated as a possible way for diagnosis and clinical research, as it is a non-invasive, low-cost, and easy-to-handle strategy. Among the electrical properties studied, electrical impedance has been employed in different pathologies, such as lung cancer [5] and spinal cord injury [6] for diagnosis. The impedance of a circuit to a current depends on the nature of biological tissues and the frequency of stimulation [7].

In the pathophysiological setting, OA alters the mechanical and histological properties of the articular cartilaginous structures at the interface and the synovial fluid characteristics, so it probably alters impedance in individuals with OA [8]. A study in individuals with knee OA using bioimpedance spectroscopy observed that impedance increases according to the intensity of the disease [9]. The results suggest that dynamic knee conditions can be used to assess arthritic status using electrical impedance.

Understanding the specificity of the effects of osteoarthritis, we considered the hypothesis of a difference related to electrical impedance parameters between the affected and unaffected hip. Therefore, the study aims to analyze the electrical bioimpedance parameters of the lower limbs of individuals with hip OA and healthy individuals.

## Methods

This was a cross-sectional study. The study was conducted according to the recommendations of The Strengthening the Reporting of Observational Studies in Epidemiology (STROBE) [10]. The project was approved by the Ethics Committee under number 2,750,698. Volunteers with osteoarthritis clinics were matched by age and recruited by electronic means. All the volunteers who participate in the research will submit an informed consent term.

The data was collected in the morning. Initially, we collected demographic and anthropometric data, and subsequently, we applied the Numeric Pain Rating Scale (NRS), WOMAC, Harris Hip Score (HHS), and bioimpedance.

### Sample

Sample calculation was performed in the Ene® software (version 3.0, Universidad Autónoma de Barcelona, Spain). The sample size was calculated based on a study by Zou et al. [11], and the calculation was based on the detection of a moderate association (r = 0.50) among variables. Considering the statistical power of 80% and alpha of 0.05, the number of 26 volunteers was estimated.

As the recruitment criteria for the osteoarthritis group (OA group), the volunteers had to be between 45 and 70 years of age, of either gender, with a clinical and radiological diagnosis of hip osteoarthritis for at least three years, unilateral involvement, or a significant complaint in one hip according to the patient’s report.

Diagnosis of hip OA was made by an orthopedic surgeon based on clinical and radiological evaluation. The Tonnis classification outlined by Hiza et al. was used [12] to describe hip OA severity.

As an inclusion criterion, volunteers were required to obtain a value of 5 or higher on the NRS during passive motion, up to the maximum limit of hip flexion, to ensure a moderate degree of pain for the participants.

The control group (C group) was in the same age range as the volunteers with OA, both genders and absence of pain complaints.

The following conditions were adopted as non-inclusion criteria: the presence of a hip prosthesis or any implant; the presence of another degenerative disease associated with osteoarthritis; the presence of systemic diseases; clinical diagnosis of fibromyalgia; and continuous use of pain medication.

### Instruments

The same evaluator performed anamnesis and evaluations. In the anamnesis, the following data were collected: body mass (kg), height (m), history of diseases, use of medications, and whether they had undergone surgery or physiotherapeutic treatment.

The NRS is a simple and easy-to-apply scale, which consists of a sequence of numbers from 0 to 10, in which 0 represents “no pain” and 10 stands for “the worst imaginable pain”. This scale was validated for the Portuguese language [13].

The WOMAC was validated and adapted for the Brazilian population, containing 24 items evaluating pain, joint stiffness, and physical function aspects. There are five response options for each question on a Likert scale (none, mild, moderate, strong, and very strong) - with scores from 0 to 4, where zero is the absence of the symptom and 4 is the worst score for that symptom. Each dimension receives a score, which is transformed into a scale from zero, the best state of health, to 100 points the worst possible form of health [14].

The HHS questionnaire was translated and adapted to the Brazilian population and was originally developed to evaluate the results of total hip arthroplasty surgery. However, it is also used to evaluate the functionality of the individual who is affected by osteoarthritis. It consists of four categories that assess pain, function (during walking, support, and how far they can walk), activities (going up and down stairs, putting on socks and shoes, sitting, and using public transportation), and whether there are deformities and range of motion. The categories pain and function carry the most weight (44 and 47 points respectively), followed by deformities (5 points) and range of motion (4 points). The final score can be as high as 100 points. A total score of fewer than 70 points is a poor score, 70 to 80 points are reasonable, 80 to 90 points are good, and 90 to 100 points are excellent [15].

Tonnis’ classification is one of the most widely used tools for the radiographic evaluation of osteoarthritis to determine the extent. It can be classified into 4 grades, being Grade 0 with no signs of arthrosis; Grade 1 mild: increased sclerosis, mild joint space narrowing, no or mild loss of head sphericity; Grade 2 moderate: small cysts, moderate joint space narrowing, moderate loss of head sphericity; Grade 3 severe: large cysts, severe joint space narrowing or obliteration, severe head deformity [12]. he tool has reasonable reliability [16].

Regarding electrical impedance, InBodyS10 segmental bioelectrical impedance analysis equipment (BioSpace, Gangnam-gu, Seoul, South Korea) was used to evaluate the electrical impedance of lower limbs. Before the examination, the volunteers remained supine for 10 min in a temperature-controlled environment (23 ± 2ºC). Eight electrodes were positioned bilaterally, two on the infrapatellar region, one on the middle finger, and one on the thumb. Data were recorded at a frequency of 50 kHz. In the study by Ching et al. [17] with individuals with chronic pain, excellent reliability was observed (intraclass correlation coefficient of 0.99).

### Statistical analysis

The statistical analyses were performed in the JAMOVI software (version 2.3, Sydney, Australia). The Kolmogorov-Smirnov test was used for data normality. The t-test was used for intragroup comparisons for parametric data (reactance, phase angle, impedance, and lean mass) and intergroup for demographic characteristics. Data were presented as mean, standard deviation, the difference between adjusted means, and 95% confidence interval of these differences. Clinical differences were tested using Cohen’s d, where values near 0.2 indicate a small effect, near 0.5 means a moderate result, and ≥ 0.8 shows a significant impact [18]. For all analyses, 5% significance was considered.

## Results

Thirty-six individuals with hip OA were recruited for the study, five of them were not included because they did not meet the pre-established criteria in the evaluation of pain intensity upon passive movement. We included 31 volunteers in the OA Group (27 men and 4 women). For C Group, 29 healthy individuals (14 men and 15 women) were recruited and included. The demographic characteristics of the study population are described in Table 1, a difference between groups was observed, with group C having a higher number of female participants compared to group OA. Furthermore, no differences were found between the groups (p > 0.05) concerning these characteristics. The HHS and WOMAC scores showed a difference (p < 0.05) between the groups, with higher values for the OA group.

**Table 1:** Anthropometric and performance characteristics of the volunteers from the Osteoarthritis (OA) and Control (C) groups

Variables	OA (n = 31)	C (n = 29)
**Sex n (%)**		
Male	27 (87.1)	14 (48.2)
Female	4 (12.9)	15 (51.7) *
**Laterality of involvement n (%)**		
Right	12 (38.7)	13 (44.8)
Left	19 (61.3)	16 (55.1)
**Age (y) Mean ± SD**	54.06 ± 6.10	52.34 ± 4.84
**Weight (kg) Mean ± SD**	77.14 ± 15.02	80.72 ± 16.27
**Height (m) Mean ± SD**	1.67 ± 0.07	1.67 ± 0.09
**BMI (kg/m** ^**2**^ **) Mean ± SD**	27.57 ± 5.12	29.00 ± 4.34
**Diagnostic time (y) Mean ± SD**	7.00 ± 3.28	-
**NRS Passive (0–10) Mean ± SD**		
Affected hip	7.52 ± 1.52	0.00 ± 0.00*
Contralateral hip	0.94 ± 2.06	0.00 ± 0.00*
**HHS (0-100) Mean ± SD**	48.16 ± 10.69	100.00 ± 0.00*
**WOMAC**	67,42 ± 19,99	00.00 ± 0.00*
**Tonnis Classification (0–3) Mean ± SD**		
Affected hip	2.55 ± 0.62	-
Contralateral hip	0.74 ± 0.89	-

BMI: Body Mass Index; NRS: Numeric Rating Scale; HHS: Harris Hip Score; * versus OA group (p < 0,05)

The comparisons intragroup are presented in Tables 2 and 3, in which the following results can be seen: in the OA group, there was a significant decrease in phase angle (PhA) and muscle mass, as well as an increase in impedance at the 50 kHz frequency on the side affected by OA when compared to the contralateral side. In the C group, there was no difference between the dominant and non-dominant sides.

**Table 2:** Intragroup comparison - Reactance, Impedance, Phase Angle and Lean Mass of the osteoarthritis group

Variable	Affected hip	Contralateral hip	MD (95%CI)	P value	Cohen d (95% CI)
Reactance	24.94 ± 5.73	24.78 ± 4,42	0.16 (-1,36 to 1.68)	0.827	0.03 (-0.31 to 0.39)
Impedance	265.75 ± 45.72	244.03 ± 38.43	21.71 (13.69 to 29.74)	< 0.001*	0.99 (0.55 to 1.42)*
Phase Angle	5.39 ± 1.14	5.94 ± 1.03	-0.54 (-0.85 to -0.23)	0.001*	-0.64 (-1.02 to -0.25)*
Lean Mass	7.39 ± 1.34	7.69 ± 1.30	-0.29 (-0.40 to -0,19)	< 0.001*	-1.04 (-1.48 to -0.60)*

CI: confidence interval; MD: mean difference. Statistically (P < 0.05) and clinically (d > 0.50) significant difference*

**Table 3:** Intragroup comparison - Reactance, Impedance, Phase Angle and Lean Mass of the control group

Variable	Dominance	No dominance	MD (95%CI)	P value	Cohen d (95% CI)
Reactance	26.88 ± 5.44	27.83 ± 5.54	-0.94 ( -1.81 to -0.07)	0.189	-0.41 (-0.79 to -0.03)
Impedance	255.25 ± 45.50	254.81 ± 43.64	0.43 (-6.30 to 7.17)	0.895	0.24 (-0.34 to 0.38)
Phase Angle	5.99 ± 0.69	6.27 ± 0.70	-0.27 (-0.47 to -0.08)	0.076	-0.53 (-0.91 to -0.13)
Lean Mass	8.09 ± 2.26	8.14 ± 2.20	-0.04 (-0.12 to 0.03)	0.247	-0.21 (-0.58 to 0.15)

CI: confidence interval; MD: mean difference. No statistical (P > 0.05) and clinical (d < 0.50) significant difference

## Discussion

In the present study, we observed that the lower limb on the side of the hip affected by osteoarthritis has lower PhA, less muscle mass, and higher impedance than the side not affected by osteoarthritis, results that were not observed in the control group.

Studies have validated this angle as a prognostic indicator in critically ill patients. It is noteworthy that PhA, related to cellular balance, has been used as a measure of disease severity, as a tool for functional evaluation, and as a general health indicator. In this regard, the literature indicates that the phase angle is associated with body muscle mass as well as handgrip strength, suggesting that it is an indirect assessment of the individual’s functional status [19].

In the study by Wada et al. [20], the authors noticed a decreasing trend of PhA according to the severity of hip osteoarthritis, which is following our findings. The possible explanation for this result is that the decrease in PhA may be related to the loss of muscle mass and reduction of intracellular water [21].

PhA is an essential marker for individuals with musculoskeletal disorders since it serves to identify functional status [19], where high values reflect better membrane integrity and cellular function; in other words, better cellular health [22].

Another point to be highlighted is that, although men predominantly formed the OA group, PhA did not show to differ between the groups since Mattiello et al. [23] found higher values for men compared to healthy women. The authors complement that in both genders, the importance of phase angles has a similar pattern that starts in infants, increases progressively until the adolescence phase, stabilizes in adulthood, and then progressively decreases in older and elderly individuals.

The results of the present study agree with the findings obtained by Gajre et al. [24] and Neves et al. [9], who observed an increase in the impedance of individuals with knee osteoarthritis compared to healthy individuals using plethysmography and spectroscopy. However, none of these studies sought to compare the possible differences between the affected limb and the non-afflicted one, where we observed a difference between the muscles of the lower limbs of the hip affected by OA and the non-affected one, unpublished results show that hypotrophy of the lower limb can explain due to disuse caused by pain or even due to arthrogenic inhibition. Thus, the stem with less muscle mass presents greater impedance due to the smaller amount of fluid [25].

It is still necessary to expand the studies involving electrical impedance to measure the effect of treatment on these variables. In a case series, it was observed that, after recovery from injury in soccer players, the values returned to baseline levels [26].

Thus, the importance of the findings of this study is related to the possibility of using electrical bioimpedance as a quantitative biological signal in the evaluation of muscle mass and cellular function in the limb affected by hip osteoarthritis, as well as in detecting the affected lower limb. Besides being a non-invasive tool, it is safe and easy to apply and can be incorporated into the routine of exams for the patient’s segment, minimizing the use of imaging exams.

The study has a limitation: the control group had a larger number of female participants, and it was not possible to pair the groups by gender. However, because no inter-group comparison was made, this did not affect the results found.

## Conclusion

The results of this study suggest that the segmental electrical bioimpedance analysis at a frequency of 50 kHz can detect differences between the affected and non-affected limbs in individuals with hip osteoarthritis, especially the phase angle, muscle mass, and impedance.

## Data Availability

The data and materials in this paper are available from the corresponding author upon request.
